# Intravenous immunoglobulin (IVIg) dampens neuronal toll-like receptor-mediated responses in ischemia

**DOI:** 10.1186/s12974-015-0294-8

**Published:** 2015-04-15

**Authors:** Ker Zhing Lok, Milan Basta, Silvia Manzanero, Thiruma V Arumugam

**Affiliations:** Department of Physiology, Yong Loo Lin School of Medicine, National University of Singapore, Block MD9, 2 Medical Drive #04-01, Singapore, 117597 Singapore; School of Biomedical Sciences, The University of Queensland, Chancellors Pl, Brisbane, QLD 4072, Australia; Biovisions, Inc., 9012 Wandering Trail Dr, Potomac, MD USA; Australian Institute for Bioengineering and Nanotechnology, The University of Queensland, Building 75, Cnr College Rd & Cooper Rd, Brisbane, QLD 4072 Australia

**Keywords:** TLRs, IVIg, Neuronal death, HMGB1, Ischemic stroke

## Abstract

**Background:**

Ischemic stroke causes a high rate of deaths and permanent neurological damage in survivors. Ischemic stroke triggers the release of damage-associated molecular patterns (DAMPs) such as high-mobility group box 1 (HMGB1), which activate toll-like receptors (TLRs) and receptor for advanced glycation endproducts (RAGE) in the affected area, leading to an exaggerated inflammatory response and cell death. Both TLRs and RAGE are transmembrane pattern recognition receptors (PRRs) that have been shown to contribute to ischemic stroke-induced brain injury. Intravenous immunoglobulin (IVIg) preparations obtained by fractionating human blood plasma are increasingly being used as an effective therapeutic agent in the treatment of several inflammatory diseases. Its use as a potential therapeutic agent for treatment of stroke has been proposed, but little is known about the direct neuroprotective mechanisms of IVIg. We therefore investigate whether IVIg exerts its beneficial effects on the outcome of neuronal injury by modulating HMGB1-induced TLR and RAGE expressions and activations.

**Methods:**

Primary cortical neurons were subjected to glucose deprivation or oxygen and glucose deprivation conditions and treated with IVIg and recombinant HMGB1. C57/BL6J mice were subjected to middle cerebral artery occlusion, followed by reperfusion, and IVIg was administered intravenously 3 h after the start of reperfusion. Expression of TLRs, RAGE and downstream signalling proteins in neurons and brain tissues were evaluated by immunoblot.

**Results:**

Treatment of cultured neurons with IVIg reduced simulated ischemia-induced TLR2, TLR4, TLR8 and RAGE expressions, pro-apoptotic caspase-3 cleavage and phosphorylation of the cell death-associated kinases such as c-Jun N-terminal kinase (JNK), p38 mitogen-activated protein kinase (MAPK) as well as the p65 subunit of nuclear factor kappa B (NF-κB). These results were recapitulated in an *in vivo* model of stroke. IVIg treatment also upregulated the anti-apoptotic protein B-cell lymphoma 2 (Bcl-2) in cortical neurons under ischemic conditions. Finally, IVIg protected neurons against HMGB1-induced neuronal cell death by modulating TLR and RAGE expressions and signalling pathways.

**Conclusions:**

Taken together, these results provide a rationale for the potential use of IVIg to target inappropriately activated components of the innate immune system following ischemic stroke.

## Background

Toll-like receptors (TLRs) are expressed in a variety of mammalian immune-related cell types and initiate signals in response to diverse pathogen-associated molecular patterns (PAMPs) [1]. The signalling pathways activated by the TLRs are broadly classified into MyD88-dependent and -independent pathways [2]. Upon receptor activation and interaction with MyD88, one or more of the toll/interleukin-1 receptor (TIR)-containing adapter proteins are recruited and MyD88 binds with the TIR domain of the receptor and phosphorylates interleukin-1 receptor-associated kinase 4 (IRAK4), which, in turn, phosphorylates IRAK1. After being phosphorylated, IRAK1 dissociates from MyD88 and interacts with TRAF6. TRAF6 forms a complex that activates downstream signalling of TLRs [1]. Major downstream signalling pathways activated by TLR engagement include the nuclear factor kappa B (NF-κB) and mitogen-activated protein kinases (MAPKs) such as c-Jun N-terminal kinase (JNK) and p38 MAPK cascades. The MyD88-independent pathway signals through the adaptor TIR-domain-containing adaptor protein inducing interferon (IFN)-β-mediated transcription factor (TRIF) [1]. TRIF interacts with TRAF3 leading to the recruitment and activation of tumour necrosis factor (TNF) receptor-associated factor-family member-associated NF-κB activator-binding kinase 1 (TBK1) and inhibitor of nuclear factor kappa-B kinase subunit epsilon (IKKε). This culminates in interferon regulatory factor 3 (IRF3) phosphorylation that facilitates IRF3 dimerization and translocation into the nucleus and transcriptional regulation. In relation to cerebral ischemia, it has been shown that disruption of downstream MyD88-independent (TRIF) TLR pathway does not confer protection in *in vitro* and *in vivo* models of cerebral ischemia [3]. A variety of endogenous ligands, such as high-mobility group box 1 (HMGB1), also bind to TLR and activate several intracellular inflammatory pathways, including the NF-κB, JNK and p38 MAPK pathways [4,5]. The presence of several TLRs has been reported in the brain, both in glial and neuronal cells [6-9], and recent studies have reported the pathological roles of TLR2, TLR4 and TLR8 in ischemic stroke-induced brain injury [7,9-12]. Neurons were found to express TLR2, TLR4 and TLR8 under both physiological and pathological conditions, and cortical neuronal cultures from both TLR2 and TLR4 deficient mice were protected against cell death induced by energy deprivation (an *in vitro* model of ischemic stroke) when compared with wild type [7]. Furthermore, we have recently provided evidence that neuronal TLR8 signalling plays a detrimental role by triggering post-stroke inflammation and neuronal cell death [9].

Intravenous immunoglobulin (IVIg) is an FDA-approved therapeutic modality used for various autoimmune and inflammatory diseases [13]. Recently, we demonstrated that IVIg treatment significantly reduced brain infarct volume and mortality in an experimental mouse model of ischemic stroke [14,15]. IVIg has also been shown to inhibit complement activation, modulate cytokine production, reduce endothelial dysfunction and death, reduce activation and infiltration of leukocytes, as well as inhibit neuronal apoptosis by decreasing the cleavage of caspase-3 in primary cortical neurons subjected to ischemic insults [14,15]. It was recently shown that IVIg may modulate TLR9 expression and activation in pathological conditions such as systemic lupus erythematosus (SLE), suggesting a new additional mechanism of IVIg [16]. IVIg has also been shown to modulate the maturation of TLR-primed peripheral blood monocytes [17]. In addition, IVIg suppresses TLR4-induced cytokine production induced by lipopolysaccharide (LPS) by inhibiting the NF-κB, JNK and p38 MAPK pathways in human monocytic cells [18]. IVIg also attenuates multiple cell death pathways by decreasing the phosphorylation of the p65 subunit of NF-κB, JNK, p38 MAPK and c-Jun, in simulated ischemic condition [15]. However, relatively little is known about the role of IVIg in modulating the expression and activation of TLRs and endogenous ligand-mediated TLR activation following ischemic stroke. Here, we provide the first evidence that IVIg protects neurons by decreasing the expression and activation of TLRs and by suppressing HMGB1-mediated TLR activation. Our findings further support IVIg as a potential therapeutic modality for targeting ischemic stroke-induced neuronal cell death and brain injury.

## Methods

### Primary cortical neuronal cultures

Dissociated neuron-enriched cell cultures of mouse cerebral cortex were established from day 16 C57BL/6 J mouse embryos, as previously described [19]. Experiments were performed in 7- to 9-day-old cultures. Approximately 95% of the cells in such cultures were neurons, and the remaining cells were astrocytes.

### Glucose deprivation and combined oxygen-glucose deprivation

For glucose deprivation (GD) studies, glucose-free Locke’s buffer containing: 154 mM NaCl, 5.6 mM KCl, 2.3 mM CaCl_2_, 1 mM MgCl_2_, 3.6 mM NaHCO_3_, 5 mM HEPES, pH 7.2, supplemented with gentamicin (5 mg/L) was used. The cultured neurons were incubated in glucose-free Locke’s buffer for up to 24 h. For combined oxygen and glucose deprivation (OGD) studies, neurons were incubated in glucose-free Locke’s buffer in an oxygen-free chamber for 4.5, 6 or 12 h. To observe the effect of IVIg, IVIg (KIOVIG, Baxter) was added at the reported concentrations to the cultures during GD or OGD conditions. The active ingredient in KIOVIG is a human plasma-derived immunoglobulin, concentration of 100 mg/mL (10% w/v), produced from large pools of human plasma by a modified Cohn-Oncley cold ethanol fractionation, yielding an intermediate immunoglobulin G, referred to as precipitate G. Equal concentration of glycine (Sigma Aldrich, St. Louis, MO, USA), an amino acid with no known affinity for any cell receptors, was also included in the experiments as vehicle control for IVIg. To observe the effect of HMGB1, mouse recombinant HMGB1 (Source: *E. coli* expressed amino acids Met1-Glu215 of mouse HMGB-1 accession # NM_010439; purity: >98%; endotoxin: less than 0.01 ng/μg cytokine) (34-8401, eBioscience, San Diego, CA, USA) was added either alone or simultaneously with IVIg to the OGD-treated neuronal cultures.

### Western blot analysis

Protein samples were subjected to sodium dodecyl sulphate-polyacrylamide (7.5% or 10%) gel electrophoresis using a Tris-glycine running buffer (Bio-Rad, Hercules, CA, USA). Gels were then electro-blotted using a semi-dry transfer apparatus (Bio-Rad) in transfer buffer containing 0.025 mol/L Tris base, 0.15 mol/L glycine and 10% (v/v) methanol for 1.5 h at 350 mA onto nitrocellulose membranes (Bio-Rad). The membranes were then incubated in blocking buffer [5% bovine serum albumin (BSA) or 5% non-fat milk in TBS-T (20 mmol/L Tris-HCl, pH 7.5, 137 mmol/L NaCl, 0.2% Tween-20)] for 1 h at room temperature. The membranes were then incubated overnight at 4°C with primary antibodies including those that selectively bind TLR2 (Abcam, Cambridge, UK), TLR4 (Abcam), TLR8 (Abcam), RAGE (Abcam), MyD88 (Abcam), TRAF6 (Abcam), p-JNK (Cell Signaling, Danvers, MA, USA), p-p38 MAPK (Cell Signaling), p-p65 (Cell Signaling), p-c-Jun (Cell Signaling), Bcl-2 (Cell Signaling), cleaved caspase-3 (Cell Signaling), HMGB1 (Cell Signaling) and β-actin (Sigma Aldrich). After washing three times (10 min per wash) with TBS-T, the membranes were incubated with horseradish peroxidase-conjugated secondary antibodies for 1 h at room temperature. The membranes were then washed again with TBS-T and incubated with chemiluminescent substrate for enhanced chemiluminescence (Thermo Fisher Scientific, Waltham, MA, USA) for 5 min. The signals were visualized by exposing the membranes onto X-ray films (Fujifilm Corporation, Minato, Tokyo, Japan).

### Middle cerebral artery occlusion and reperfusion

12- to 14-week-old C57BL/6 J male mice were used for *in vivo* experiments. The focal cerebral ischemia/reperfusion (I/R) model was similar to that described previously [20]. Briefly, the mice were anesthetized with isoflurane, a midline incision was made in the neck, and the left external carotid and pterygopalatine arteries were isolated and ligated with 6-0 silk thread. The internal carotid artery (ICA) was occluded at the peripheral site of the bifurcation of the ICA and the pterygopalatine artery using a small clip, and the common carotid artery (CCA) was ligated with 6-0 silk thread. The external carotid artery (ECA) was cut, and a 6-0 nylon monofilament with a blunted tip (0.2 to 0.22 mm) with a coagulator was inserted into the ECA. After the clip at the ICA was removed, the nylon monofilament was advanced into the origin of the middle cerebral artery (MCA) until light resistance was felt. The nylon monofilament and the CCA ligature were removed after 1 h of occlusion to initiate reperfusion. In the sham-operated group, these arteries were visualized but not disturbed. Mice were administered with 1 g/kg body weight of IVIg or vehicle by infusion into the femoral vein (approximately 250 μL) 3 h after the start of reperfusion period. Cerebral blood flow was measured by placing the animal’s head in a fixed frame after it had been anesthetized and prepared for surgery. A craniotomy was performed to access the left middle cerebral artery and was extended to allow positioning of a 0.5-mm Doppler probe (Moor LAB, Moor Instruments, Devon, UK) over the underlying parietal cortex approximately 1 mm posterior to the bregma and 1 mm lateral to the midline. The animals were included in the study if they underwent successful MCA occlusion, defined by an 80% or greater drop in cerebral blood flow seen with laser Doppler flowmetry. The animals were excluded if insertion of the thread resulted in perforation of the vessel wall determined by the presence of sub-arachnoid blood at the time of sacrifice. University of Queensland Animal Care and Use Committee approved all *in vivo* experimental procedures.

### Statistical analysis

All numerical values are expressed as mean ± SEM. The overall significance of the data was examined by one-way analysis of variance (ANOVA) followed by Newman-Keuls *post hoc* test to determine group differences. Statistical difference was considered as *P* < 0.05 throughout the study. Statistical analyses were performed using GraphPad Prism software.

## Results

### IVIg treatment reduces expression and activation of TLRs in primary cortical neurons subjected to GD and OGD conditions

**Figure 1:**
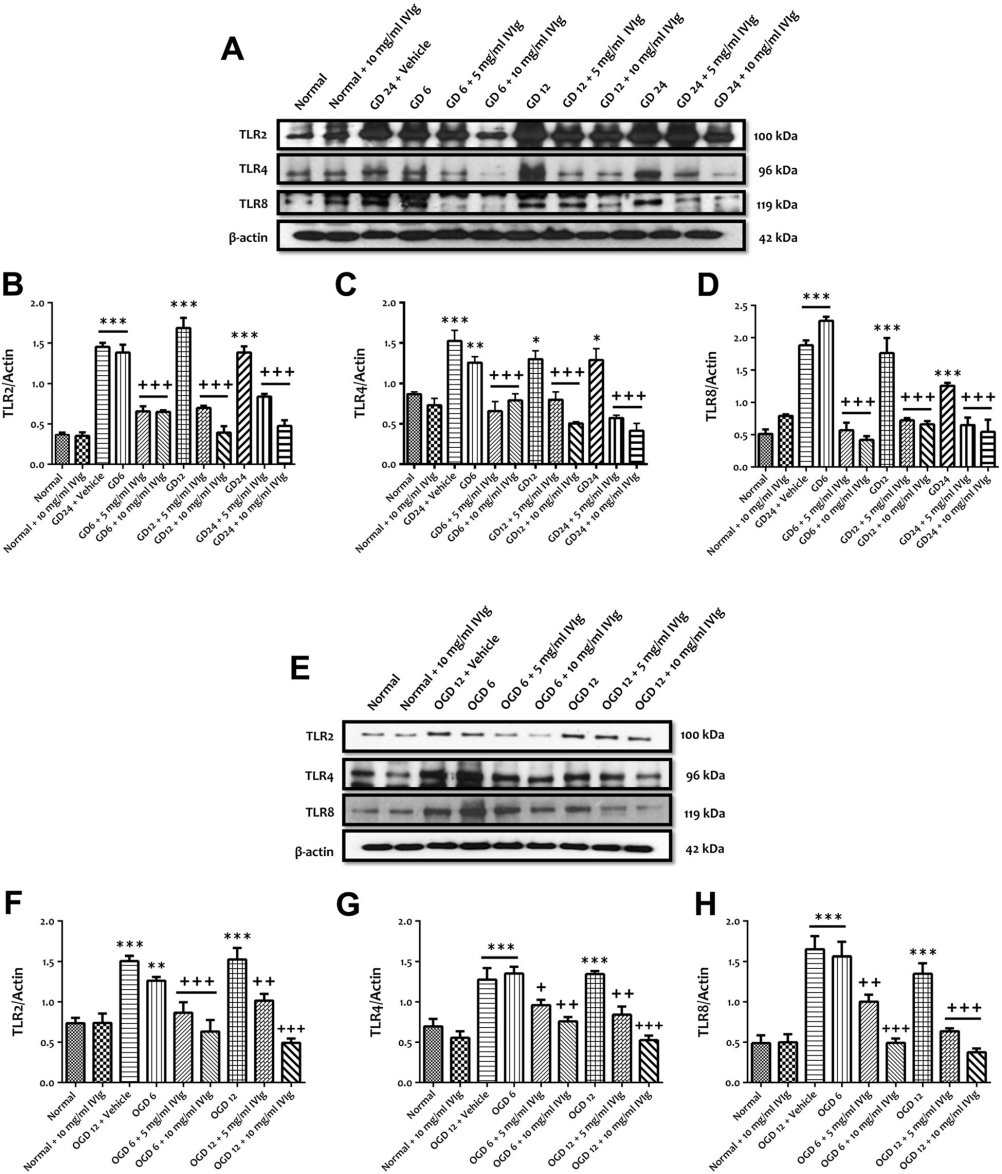
IVIg treatment inhibits the increased expressions of TLRs in cultured cortical neurons subjected to simulated ischemia. **(A-D)** Representative 2immunoblots and quantification illustrating increases in the levels of TLR2, TLR4 and TLR8 proteins in primary cortical neurons at indicated times during GD. Administration of IVIg (5 and 10 mg/ml) significantly prevents GD-induced increased expression of TLR2, TLR4 and TLR8. Data are represented as mean ± SEM. *n* = 5 cultures. ^*^ *P* < 0.05 in comparison with normal; ^**^ *P* < 0.01 in comparison with normal; ^***^ *P* < 0.001 in comparison with normal; ^+++^ *P* < 0.001 in comparison with vehicle-treated group. **(E-H)** Representative immunoblots and quantification illustrating increases in the levels of TLR2, TLR4 and TLR8 proteins in primary cortical neurons at indicated times during OGD. Administration of IVIg (5 and 10 mg/ml) significantly prevents OGD-induced increased expression of TLR2, TLR4 and TLR8. Data are represented as mean ± SEM. *n* = 5 cultures. ^**^ *P* < 0.01 in comparison with normal; ^***^ *P* < 0.001 in comparison with normal; ^+^ *P* < 0.05 in comparison with vehicle-treated group; ^++^ *P* < 0.01 in comparison with vehicle-treated group; ^+++^ *P* < 0.001 in comparison with vehicle-treated group.

**Figure 2:**
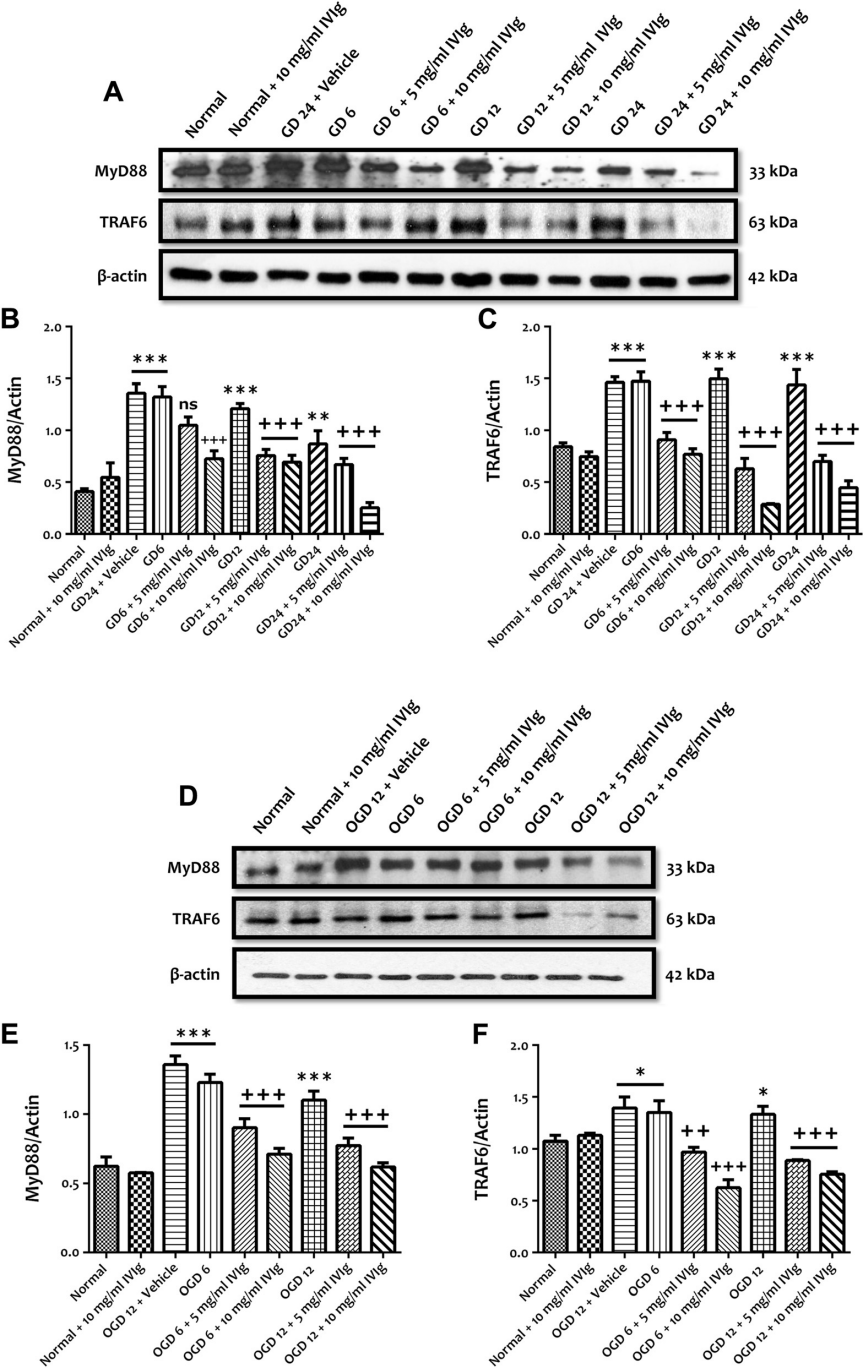
IVIg treatment prevents the increased expressions of TLR adaptor and signalling proteins. IVIg treatment prevents the increased expressions of TLR adaptor and signalling proteins in cultured cortical neurons subjected to simulated ischemia. **(A-C)** Representative immunoblots and quantification illustrating increases in the levels of MyD88 and TRAF6 proteins in primary cortical neurons at indicated times during GD. Administration of 10 mg/ml of IVIg significantly prevents the increase in MyD88 and TRAF6 protein expression caused by 6, 12 and 24 h of GD; the effect of 5 mg/ml IVIg was significant at 12 and 24, but not 6 h of GD. Data are represented as mean ± SEM. *n* = 5 cultures. ^**^ *P* < 0.01 in comparison with normal; ^***^ *P* < 0.001 in comparison with normal; ^+++^ *P* < 0.001 in comparison with vehicle-treated group; ns, not significant. **(D-F)** Representative immunoblots and quantification illustrating increases in the levels of MyD88 and TRAF6 proteins in primary cortical neurons at indicated times during OGD. Administration of IVIg (5 and 10 mg/ml) significantly prevents an increased expression level of MyD88 and TRAF6 proteins following 6 and 12 h of OGD. Data are represented as mean ± SEM. *n* = 5 cultures. ^*^ *P* < 0.05 in comparison with normal; ^***^ *P* < 0.001 in comparison with normal; ^++^ *P* < 0.01 in comparison with vehicle-treated group; ^+++^ *P* < 0.001 in comparison with vehicle-treated group.

We first evaluated the effect of IVIg on the protein expressions of TLR2, TLR4 and TLR8 in a cell culture model of ischemic neuronal injury in which primary mouse cortical neurons were subjected to conditions mimicking ischemic stroke, including glucose deprivation (GD) and combined oxygen and glucose deprivation (OGD) (Figure 1). We have previously shown that low concentrations (0.1 and 0.3 mg/ml) of IVIg significantly increased *in vitro* ischemic stress-induced neuronal cell death whereas high concentrations (5 and 10 mg/ml) of IVIg significantly decreased GD-induced neuronal cell death [15]. Therefore, we used 5 and 10 mg/ml in this study to test the effect of IVIg against TLR expression and activation. Immunoblot analyses showed that treatment with IVIg (5 and 10 mg/ml) significantly prevented GD-induced increased expressions of TLR2, TLR4 and TLR8 following 6, 12 and 24 h (Figure 1A-D). Treatment with IVIg (5 and 10 mg/ml) also significantly prevented 6 and 12 h OGD-induced increased neuronal expressions of TLR2, TLR4 and TLR8 (Figure 1E**-**H). We next analysed the effect of IVIg on the expression levels of myeloid differentiation primary response gene (88) (MyD88) and TNF receptor-associated factor 6 (TRAF6) following GD and OGD conditions. Both of these proteins are involved in the downstream signalling of TLR2, TLR4 and TLR8. Our immunoblot analyses showed that GD- and OGD-induced upregulation of MyD88 and TRAF6 levels at different time points was significantly prevented by IVIg treatment (Figure 2A**-**F).

### IVIg modulates neuronal NF-κB and MAPK activities and protects neurons against apoptotic cell death following simulated ischemic condition

**Figure 3:**
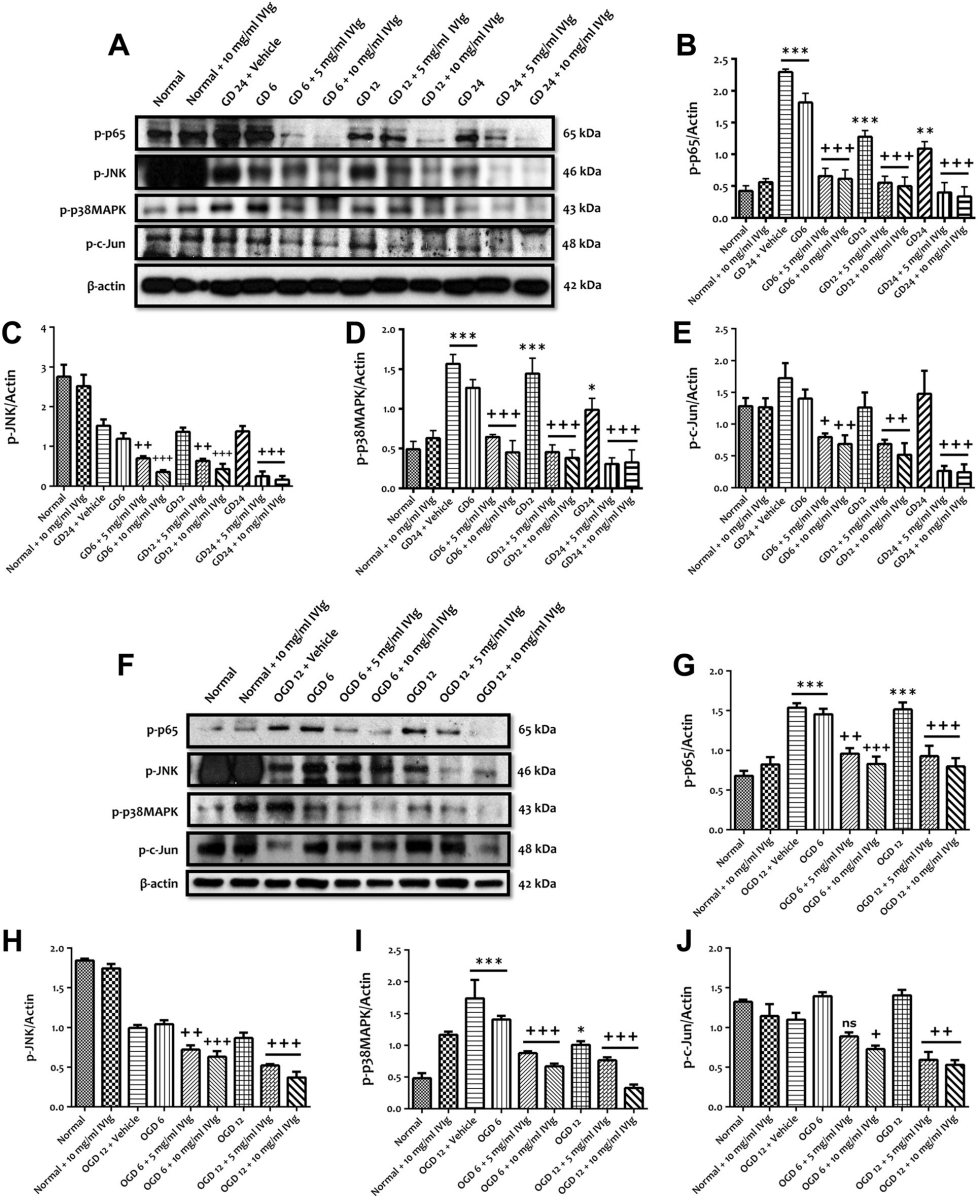
IVIg treatment down regulates the expressions of the phosphorylated forms of TLR signalling cascade components. IVIg treatment down regulates the expressions of the phosphorylated forms of TLR signalling cascade components in cultured cortical neurons subjected to simulated ischemia. **(A-E)** Representative immunoblots and quantification illustrating increases in the levels of NF-κB-p-p65 and p-p38 MAPK in primary cortical neurons at indicated times during GD. Administration of IVIg (5 and 10 mg/ml) significantly attenuated the expression level of NF-κB-p-p65, p-JNK, p-p38 MAPK and p-c-Jun in GD 6-, 12- and 24-h-treated neurons. Data are represented as mean ± SEM. *n* = 5 cultures. ^*^ *P* < 0.05 in comparison with normal; ^**^ *P* < 0.01 in comparison with normal; ^***^ *P* < 0.001 in comparison with normal; ^+^ *P* < 0.05 in comparison with vehicle-treated group; ^++^ *P* < 0.01 in comparison with vehicle-treated group; ^+++^ *P* < 0.001 in comparison with vehicle-treated group. **(F-J)** Representative immunoblots and quantification illustrating increases in the levels of NF-κB-p-p65 and p-p38 MAPK in primary cortical neurons at indicated times during OGD. Administration of IVIg (5 and 10 mg/ml) significantly attenuated the expression level of NF-κB-p-p65, p-JNK and p-p38 MAPK in OGD 6- and 12-h-treated neurons. Administration of 10 mg/ml IVIg significantly attenuated the expression level of p-c-Jun in OGD 6- and 12-h-treated neurons, however the effect of 5 mg/ml IVIg was significant at 12 but not 6 h of OGD. Data are represented as mean ± SEM. *n* = 5 cultures. ^*^ *P* < 0.05 in comparison with normal; ^***^ *P* < 0.001 in comparison with normal; ^+^ *P* < 0.05 in comparison with vehicle-treated group; ^++^ *P* < 0.01 in comparison with vehicle-treated group; ^+++^ *P* < 0.001 in comparison with vehicle-treated group; ns, not significant.

**Figure 4:**
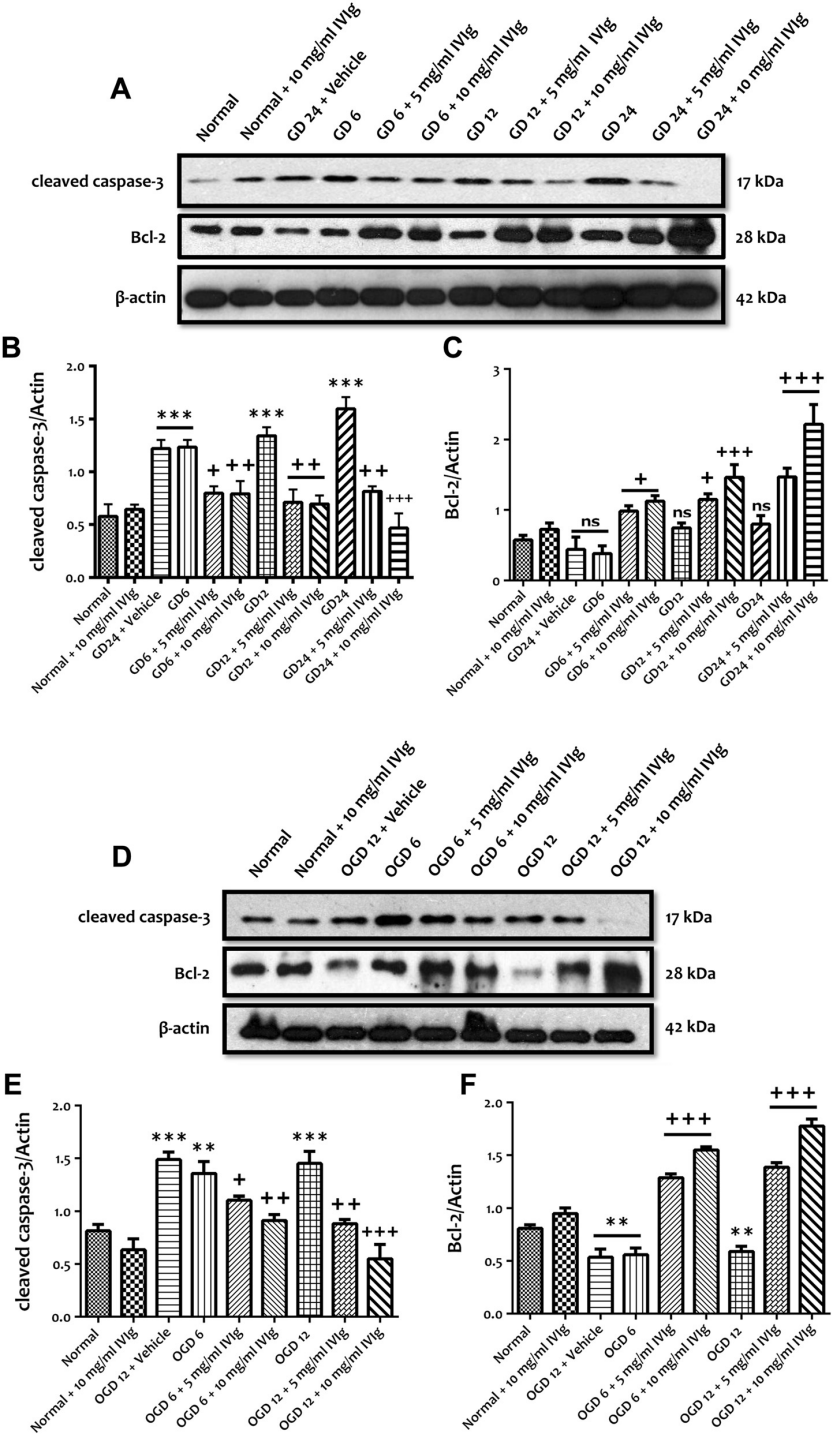
IVIg protects cultured neurons against simulated ischemia. **(A, B)** Treatment with high concentrations of IVIg (5 and 10 mg/ml) significantly prevents GD-induced increased expression of pro-apoptotic cleaved caspase-3 following 6, 12 and 24 h. Data are represented as mean ± SEM. *n* = 5 cultures. ^***^ *P* < 0.001 in comparison with normal; ^+^ *P* < 0.05 in comparison with vehicle-treated group; ^++^ *P* < 0.01 in comparison with vehicle-treated group; ^+++^ *P* < 0.001 in comparison with vehicle-treated group. **(A, C)** Treatment with high concentrations of IVIg (5 and 10 mg/mL) significantly increased the expression level of anti-apoptotic Bcl-2 following 6, 12 and 24 h of GD. Data are represented as mean ± SEM. *n* = 5 cultures. ^+^ *P* < 0.05 in comparison with vehicle-treated group; ^+++^ *P* < 0.001 in comparison with vehicle-treated group; ns, not significant. **(D, E)** Treatment with high concentrations of IVIg (5 and 10 mg/mL) significantly prevents OGD-induced increased expression of pro-apoptotic cleaved caspase-3 following 6 and 12 h. Data are represented as mean ± SEM. *n* = 5 cultures. ^**^ *P* < 0.01 in comparison with normal; ^***^ *P* < 0.001 in comparison with normal; ^+^ *P* < 0.05 in comparison with vehicle-treated group; ^++^ *P* < 0.01 in comparison with vehicle-treated group; ^+++^ *P* < 0.001 in comparison with vehicle-treated group. **(D, F)** Treatment with high concentrations of IVIg (5 and 10 mg/mL) significantly prevents a decreased level of anti-apoptotic Bcl-2 following 6 and 12 h of OGD. Data are represented as mean ± SEM. *n* = 5 cultures. ^**^ *P* < 0.01 in comparison with normal; ^+++^ *P* < 0.001 in comparison with vehicle-treated group.

Because the activation of NF-κB, JNK and p38 MAPK pathways are implicated in TLR2, TLR4 and TLR8 signalling and ischemia-induced neuronal cell death [10,21], we next measured the protein levels of phosphorylated p65 (a subunit of NF-κB), JNK, p38 MAPK and c-Jun following GD and OGD in IVIg-treated neurons compared with control neurons. Treatment with both 5 and 10 mg/ml of IVIg significantly attenuated the expression level of the phosphorylated forms of NF-κB p65 (Figure 3A,B), JNK, p38 MAPK and c-Jun following 6, 12 and 24 h of GD when compared to the vehicle-treated group (Figure 3A,C-E). We also measured the levels of these proteins under OGD conditions. Immunoblot analyses showed that IVIg treatments attenuated the expression level of the phosphorylated forms of NF-κB p65, JNK, p38 MAPK and c-Jun following 6 and 12 h of OGD, however statistical significance was not reached for the expression level of phosphorylated c-Jun in neurons treated with 5 mg/ml of IVIg following 6 h of OGD (Figure 3F**-**J). Consistent with our previous findings, the immunoblot results showed that IVIg treatment significantly prevented an increased expression of cleaved caspase-3 and significantly increased the expression of Bcl-2 in primary cortical neurons following 6, 12 and 24 h of GD (Figure 4A**-**C). Similarly, we have also observed that IVIg treatment significantly prevented an increased expression of cleaved caspase-3 and significantly prevented a decreased expression of Bcl-2 in OGD 6- and 12-h-treated neurons (Figure 4D**-**F). These results not only confirm our previously published data following GD conditions but also show for the first time that IVIg can protect neurons following OGD conditions by downregulating apoptosis and upregulating the anti-apoptotic protein, Bcl-2.

### IVIg reduces the expression and activation of TLRs as well as NF-κB and MAPK activities *in vivo* following stroke

**Figure 5:**
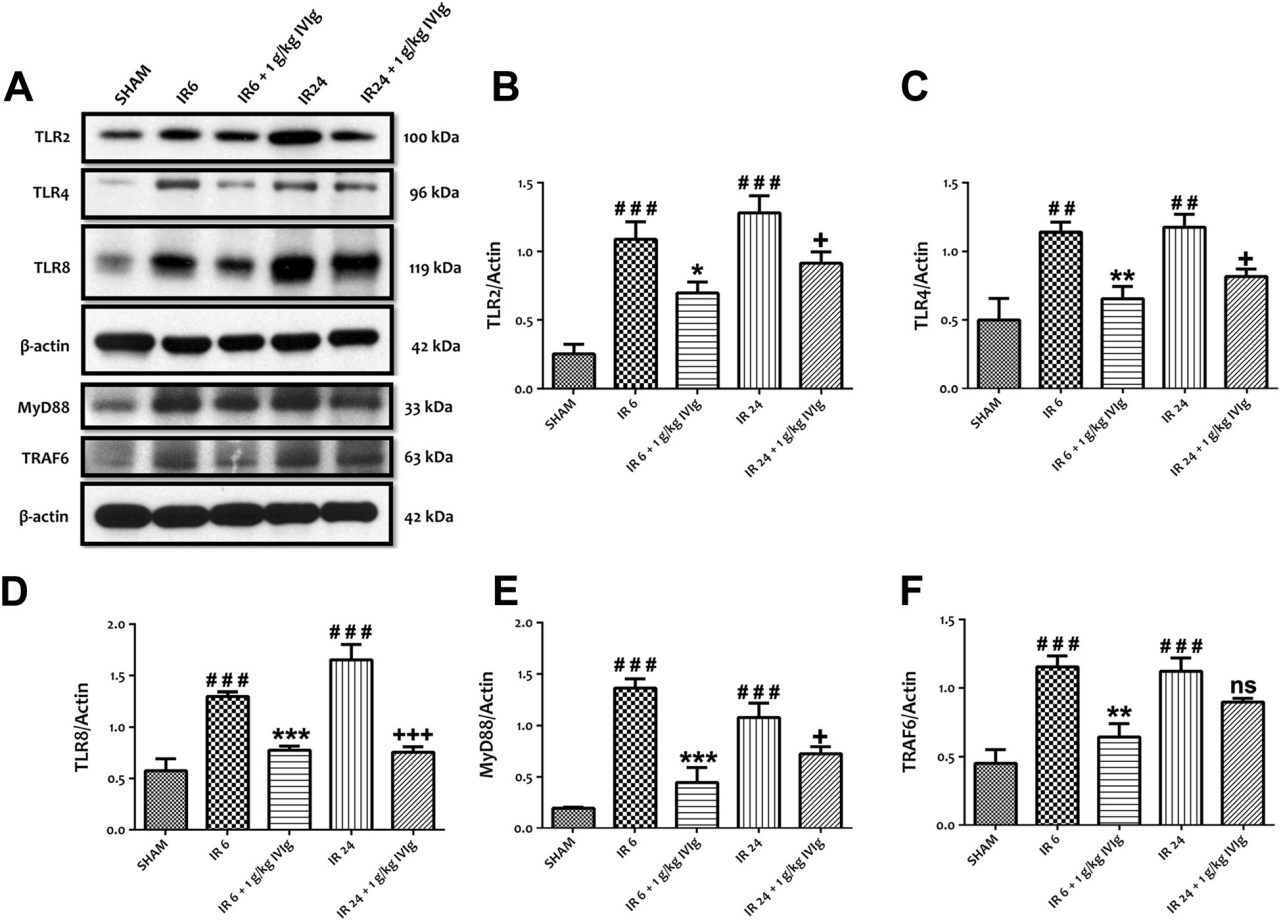
IVIg treatment prevents the increased expression of TLR signalling components *in vivo* following stroke. **(A-F)** Representative immunoblots and quantification illustrating increases in the levels of TLR2, TLR4 and TLR8 proteins and both TLR adaptor protein MyD88 and signalling protein TRAF6 in ipsilateral brain tissues of C57BL/6 J mice following MCAO (1 h) and reperfusion (6 and 24 h). Administration of IVIg (1 g/kg) significantly prevents the increased expression levels of TLR2, TLR4 and TLR8 as well as MyD88 following 6 and 24 h of cerebral ischemia and reperfusion (I/R). However, administration of IVIg (1 g/kg) only significantly prevents the increased expression level of TRAF6 in mice subjected to 6 h of cerebral I/R, not in mice subjected to 24 h of cerebral I/R. Data are represented as mean ± SEM. *n* = 5 to 6 animals in each group. ^##^ *P* < 0.01 in comparison with sham; ^###^ *P* < 0.001 in comparison with sham; *P < 0.05 in comparison with I/R 6; **P < 0.01 in comparison with I/R 6; ***P < 0.001 in comparison with I/R 6; ^+^ *P* < 0.05 in comparison with I/R 24; ^+++^ *P* < 0.001 in comparison with I/R24; ns, not significant.

**Figure 6:**
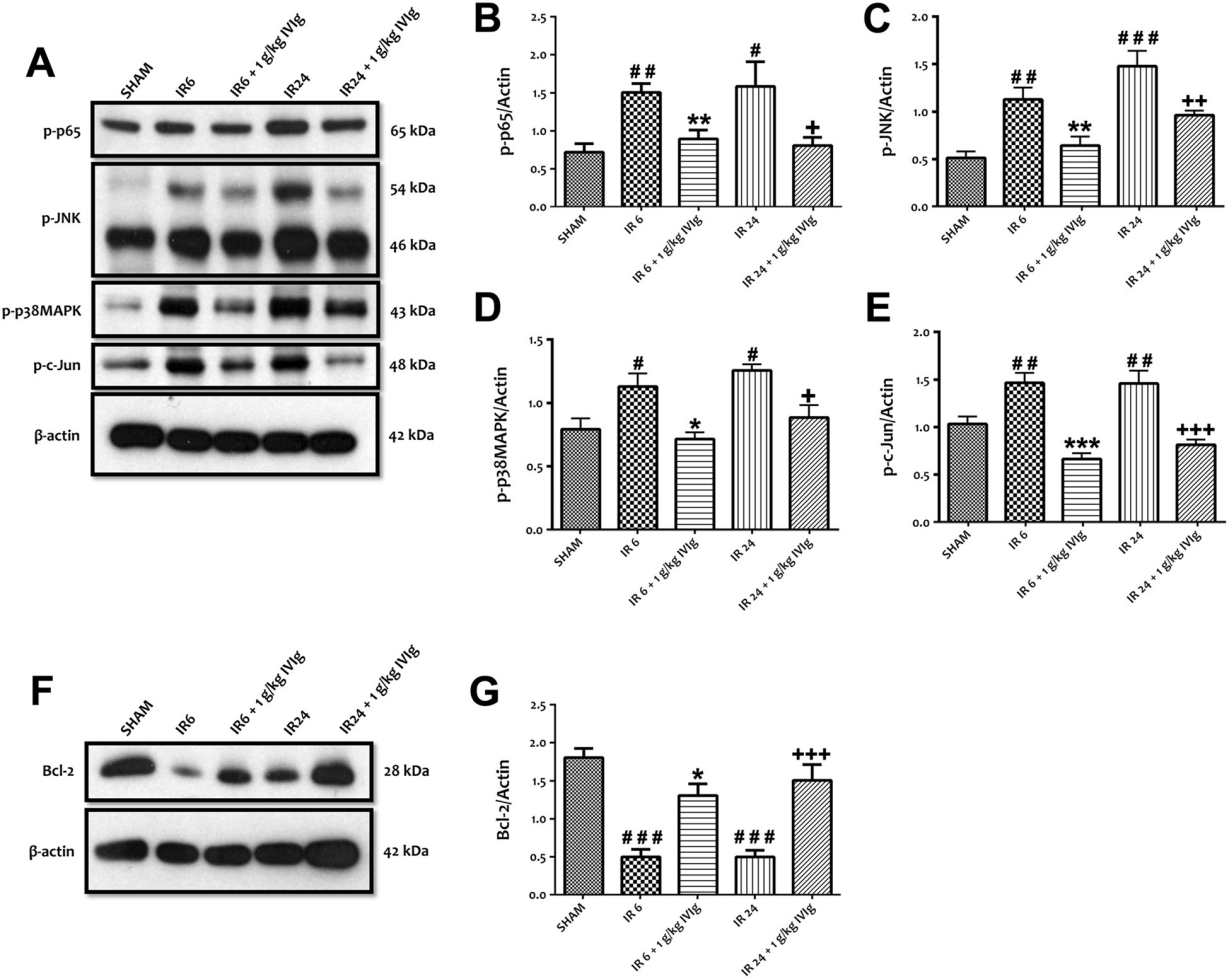
IVIg treatment prevents an increased expression level of the phosphorylated forms of TLR signalling cascade components. IVIg treatment prevents an increased expression level of the phosphorylated forms of TLR signalling cascade components *in vivo* following stroke. **(A-E)** Representative immunoblots and quantification illustrating increases in the levels of NF-κB-p-p65, p-JNK, p-p38 MAPK and p-c-Jun in ipsilateral brain tissues of C57BL/6 J mice following MCAO (1 h) and reperfusion (6 and 24 h). Administration of IVIg (1 g/kg) significantly prevents the increased expression levels of NF-κB-p-p65, p-JNK, p-p38 MAPK and p-c-Jun following stroke. Data are represented as mean ± SEM. *n* = 5 to 6 animals in each group. ^#^ *P* < 0.05 in comparison with sham; ^##^ *P* < 0.01 in comparison with sham; ^###^ *P* < 0.001 in comparison with sham; *P < 0.05 in comparison with I/R 6; **P < 0.01 in comparison with I/R 6; ***P < 0.001 in comparison with I/R 6; ^+^ *P* < 0.05 in comparison with I/R 24; ^++^ *P* < 0.01 in comparison with I/R 24; ^+++^ *P* < 0.001 in comparison with I/R 24. **(F, G)** Representative immunoblots and quantification illustrating decrease in the levels of anti-apoptotic Bcl-2 in ipsilateral brain tissues of C57BL/6 J mice following MCAO (1 h) and reperfusion (6 and 24 h). Administration of IVIg (1 g/kg) significantly prevents a decreased level of Bcl-2 following stroke. Data are represented as mean ± SEM. n = 5 to 6 animals in each group. ^###^ *P* < 0.001 in comparison with sham; *P < 0.05 in comparison with I/R 6; ^+++^ *P* < 0.001 in comparison with I/R 24.

We previously reported that pre- and post-treatment with IVIg reduced brain damage and neurological deficits after cerebral I/R in mice [14]. However, we do not know if the IVIg-mediated protective effect *in vivo* is partly achieved by modulating TLR expression and activation. Therefore, we next analysed the expression of TLR2, TLR4 and TLR8 *in vivo* following cerebral I/R injury. Our data showed that 6 and 24 h of cerebral I/R-induced increased expressions of TL2, TLR4 and TLR8 were significantly prevented by post-treatment with 1 g/kg body weight of IVIg 3 h after the onset of reperfusion (Figure 5A**-**D). We next analysed the expression levels of TLR adaptor and signalling proteins, MyD88 and TRAF6 following cerebral I/R. Similar to the *in vitro* findings, we observed that 3-h post-treatment with 1 g/kg of IVIg significantly prevented an increased expression level of MyD88 following 6- and 24-h cerebral I/R injury (Figure 5A,E). A 1 g/kg of IVIg treatment significantly prevented an increased expression level of TRAF6 following 6-h cerebral I/R injury, however statistical significance was not reached following 24-h cerebral I/R injury (Figure 5A,F). We next analysed the activity of TLR downstream signalling proteins such as NF-κB and MAPKs *in vivo* following cerebral I/R injury. IVIg treatment significantly prevented an increased expression level of phosphorylated NF-κB p65 (Figure 6A,B), JNK, p38 MAPK and c-Jun following 6- and 24-h cerebral I/R as compared with control group (Figure 6A,C**-**E). Immunoblot analyses showed that IVIg treatment significantly prevented a decreased level of Bcl-2 following both 6- and 24-h cerebral I/R as compared with control group (Figure 6F,G).

### IVIg protects neurons against HMGB1-induced cell death by modulating TLR signalling

**Figure 7:**
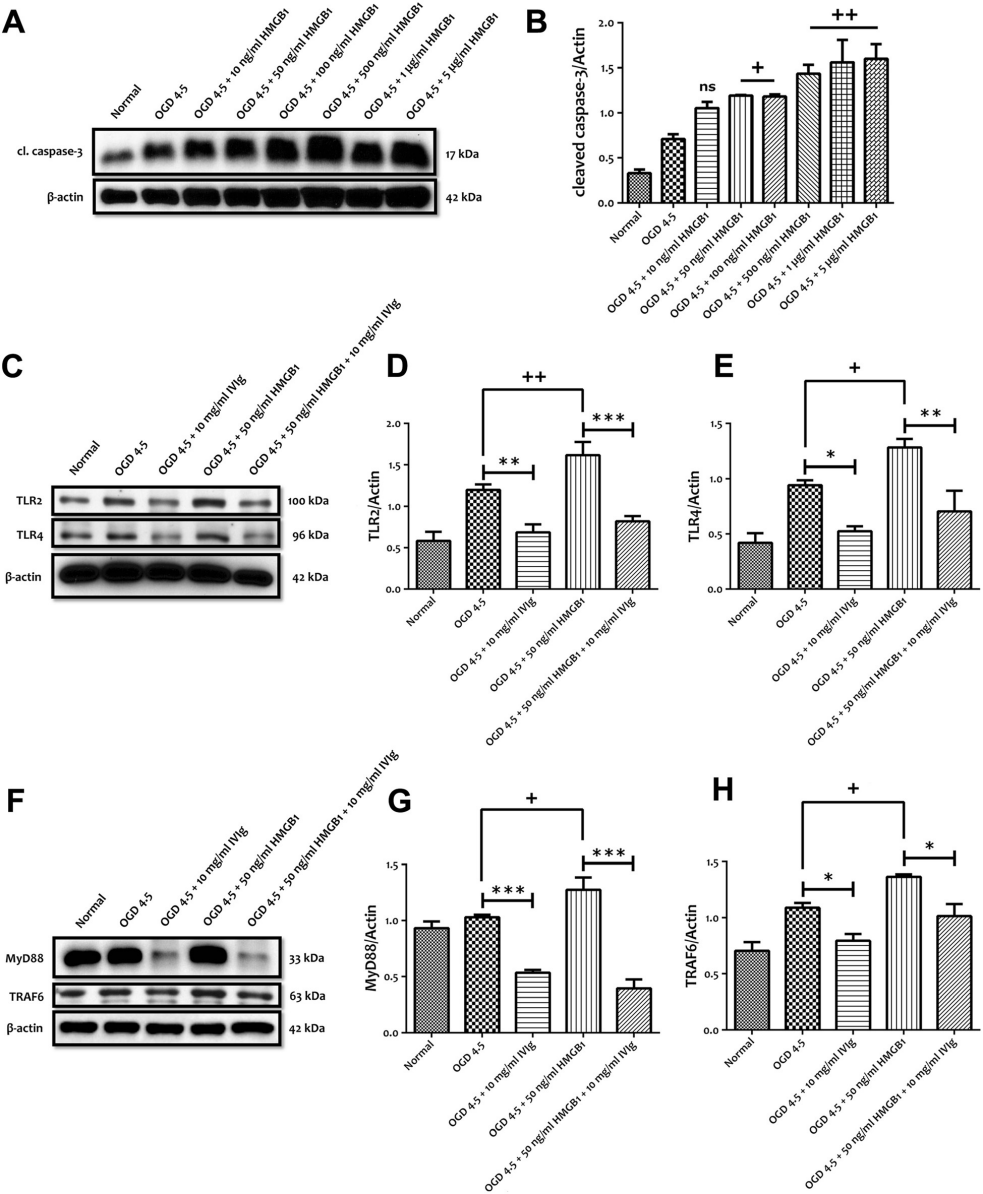
IVIg protects primary neurons against HMGB1-induced cell death by modulating TLRs expression. **(A, B)** Different concentrations of mouse recombinant HMGB1 significantly increase oxygen and glucose deprivation (OGD)-induced pro-apoptotic cleaved caspase-3 level. Data are represented as mean ± SEM. *n* = 3 cultures. ^+^ *P* < 0.05 in comparison with OGD 4.5-h group; ^++^ *P* < 0.01 in comparison with OGD 4.5-h group; ns, not significant. **(C-E)** Representative immunoblots and quantification illustrating HMGB1 increases the levels of TLR2 and TLR4 in primary cortical neurons following 4.5 h of OGD. Administration of IVIg (10 mg/ml) significantly prevents HMGB1-induced increased expression level of TLR2 and TLR4. Data are represented as mean ± SEM. *n* = 5 cultures. ^+^ *P* < 0.05 in comparison with OGD 4.5-h group; ^++^ *P* < 0.01 in comparison with OGD 4.5-h group; *P < 0.05 in comparison with corresponding OGD group; **P < 0.01 in comparison with corresponding OGD group; ***P < 0.001 in comparison with corresponding OGD group. **(F-H)** Representative immunoblots and quantification illustrating HMGB1 increases the levels of MyD88 and TRAF6 in primary cortical neurons following 4.5 h of OGD. Administration of IVIg (10 mg/ml) significantly prevents HMGB1-induced increased expression level of MyD88 and TRAF6. Data are represented as mean ± SEM. *n* = 5 cultures. ^+^ *P* < 0.05 in comparison with OGD 4.5-h group; *P < 0.05 in comparison with corresponding OGD group; ***P < 0.001 in comparison with corresponding OGD group.

**Figure 8:**
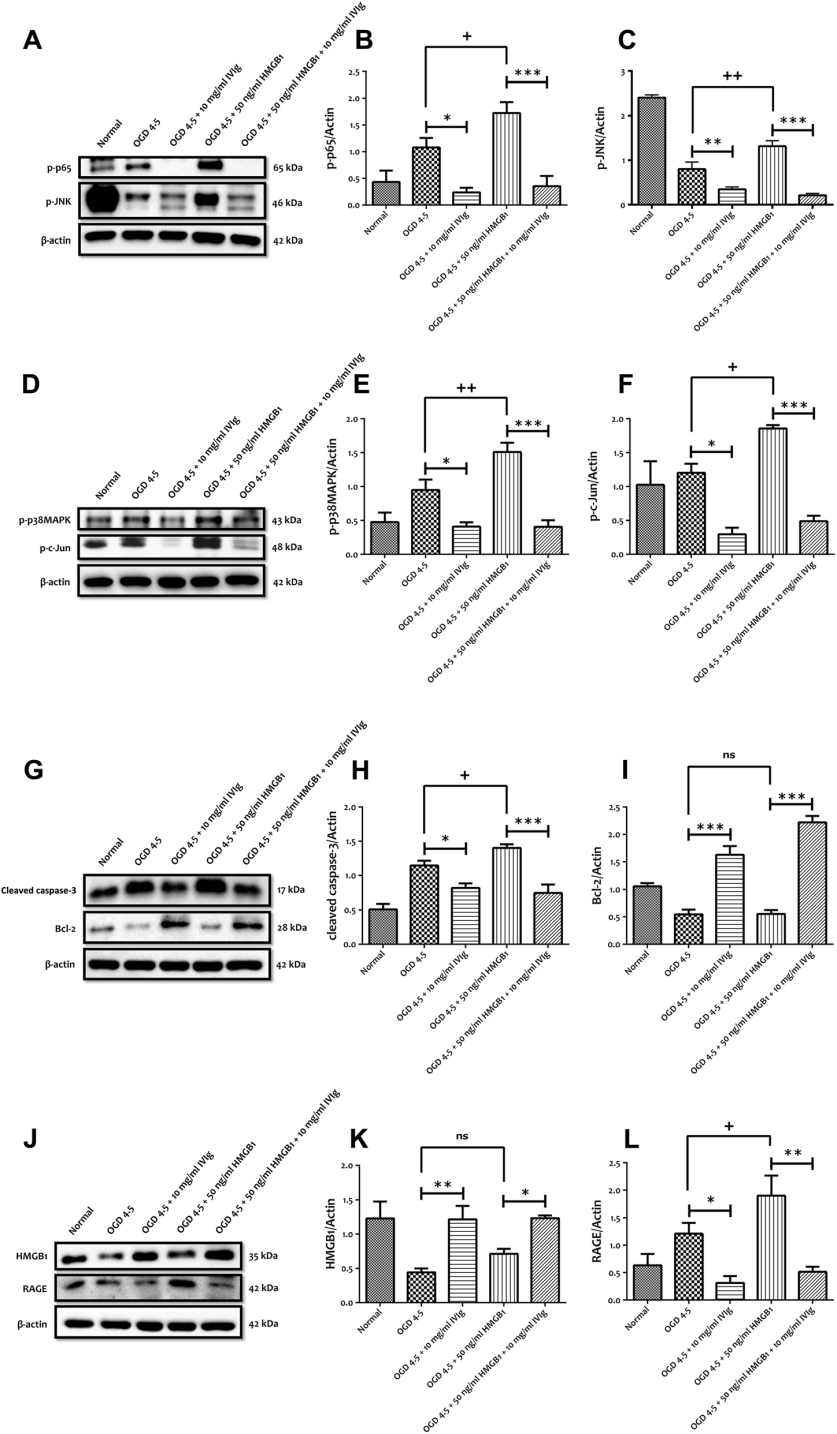
IVIg protects primary neurons against HMGB1-induced cell death by modulating TLR signalling cascades. **(A-F)** Representative immunoblots and quantification illustrating HMGB1 increases the levels of NF-κB-p-p65, p-JNK, p-p38 MAPK and p-c-Jun in primary cortical neurons following 4.5 h of OGD. The increased expression level of p-p65, p-JNK, p-p38 MAPK and p-c-Jun is significantly prevents by administration of IVIg (10 mg/ml) following HMGB1 treatment. Data are represented as mean ± SEM. *n* = 5 cultures. ^+^ *P* < 0.05 in comparison with OGD 4.5-h group; ^++^ *P* < 0.01 in comparison with OGD 4.5-h group; *P < 0.05 in comparison with corresponding OGD group; **P < 0.01 in comparison with corresponding OGD group; ***P < 0.001 in comparison with corresponding OGD group. **(G-I)** Representative immunoblots and quantification illustrating HMGB1 significantly increases the levels of pro-apoptotic cleaved caspase-3 in primary cortical neurons following 4.5 h of OGD. Administration of IVIg (10 mg/ml) significantly prevents the increased expression levels of cleaved caspase-3 and significantly prevents the decreased expression levels of Bcl-2 following HMGB1 treatment. Data are represented as mean ± SEM. *n* = 5 cultures. ^+^ *P* < 0.05 in comparison with OGD 4.5-h group; *P < 0.05 in comparison with corresponding OGD group; ***P < 0.001 in comparison with corresponding OGD group; ns, not significant. **(J-L)** Representative immunoblots and quantification illustrating the effect of IVIg on the maintenance of intracellular HMGB1 levels and the effect of HMGB1 treatment on the increase in RAGE levels. Administration of IVIg (10 mg/ml) following HMGB1 treatment was able to significantly prevent HMGB1-induced increased of RAGE expression. Data are represented as mean ± SEM. *n* = 5 cultures. ^+^ *P* < 0.05 in comparison with OGD 4.5-h group; *P < 0.05 in comparison with corresponding OGD group; **P < 0.01 in comparison with corresponding OGD group; ns, not significant.

High-mobility group box 1 (HMGB1) is a nuclear protein and has different roles, both intracellularly and extracellularly. Nuclear HMGB1 regulates chromatin structure and gene transcription whereas extracellular HMGB1 has shown to bind to receptor for advanced glycation endproducts (RAGE) and the TLRs [22,23]. In order to investigate the effect of IVIg against HMGB1-mediated TLR signalling in neurons following OGD, we first determined the effective concentration of mouse recombinant HMGB1. Our data showed that HMGB1 above 50 ng/ml was capable of significantly increasing neuronal cleavage of caspase-3 after 4.5 h of OGD (Figure 7A,B). We next analysed the effect of IVIg against HMGB1-mediated TLR expression following OGD. Our data showed that both TLR2 and TLR4 expression levels were significantly increased following HMGB1 treatment when compared to OGD alone (Figure 7C**-**E). Furthermore, HMGB1-induced increase in TLR2 and TLR4 expressions was significantly prevented by 10 mg/ml of IVIg treatment (Figure 7C**-**E). Similarly, the levels of MyD88 and TRAF6 were significantly increased with the addition of HMGB1 to OGD cultures and IVIg significantly prevented HMGB1-induced increase in MyD88 and TRAF6 following OGD condition (Figure 7F**-**H). Next, we analysed HMGB1-mediated TLR signalling pathways following IVIg treatment. Our data showed that the levels of NF-κB-p-p65, p-JNK, p38 MAPK and p-c-Jun expressions were significantly increased following HMGB1 treatment when compared to OGD alone (Figure 8A**-**F). IVIg significantly prevented HMGB1-induced increases of these proteins following OGD condition (Figure 8A**-**F). In addition, our data showed that OGD-induced cleaved caspase-3 level was significantly increased in the presence of HMGB1 and this increase was significantly prevented with IVIg treatment (Figure 8G,H). Furthermore, the decreased expression level of Bcl-2 was also prevented by IVIg treatment following HMGB1 treatment (Figure 8G,I). Finally, we analysed the total cellular HMGB1 and RAGE expression levels. Our data showed that IVIg treatment resulted in preserving HMGB1 level (Figure 8J,K). Similar to TLRs, RAGE expression level was significantly increased following HMGB1 treatments compared to OGD alone (Figure 8J,L) and HMGB1-induced increase in RAGE expression was significantly prevented by 10 mg/ml of IVIg treatment (Figure 8J,L).

## Discussion

IVIg therapy has been shown to be effective in the treatment of various inflammatory and autoimmune disorders [24]. Several mechanisms have been proposed to explain these beneficial effects of IVIg such as attenuating the activity of complement system, the expression of adhesion molecules, the expression and activation of inflammasome pathways and cytokine production [14,25,26]. IVIg acts also by dampening pro-inflammatory and apoptotic gene expressions [15,27]. We and others have previously demonstrated that IVIg treatment reduced brain damage, neurological deficit and mortality in experimental rodent models of stroke [14,15,28]. The current study reveals that a reduction in TLR expression and activation partly accounts for the protective effect of IVIg against neuronal cell death following ischemic stroke. The data indicate that IVIg reduces the expression levels of TLR2, TLR4 and TLR8 and dampens downstream signalling cascades associated with TLRs such as the NF-κB and MAPKs pathways. Furthermore, our data show that IVIg is also targeting HMGB1-mediated TLR signalling in OGD-injured neurons.

Numerous studies have reported pathological roles of TLRs and RAGE in ischemic stroke-induced brain injury [7,9,23]. We previously reported that neurons express several TLRs, and that the levels of TLR2, TLR4 and TLR8 are increased in neurons in response to ischemic conditions [7,9]. Neurons from both TLR2 knockout and TLR4-deficient mice were protected against ischemia, and the amount of brain damage and neurological deficits caused by a stroke were significantly lesser in mice deficient in TLR2 or TLR4 compared with control mice [7]. High concentrations of IVIg prevent neuronal death, but the neuroprotective mechanism is not well established. Recent studies have indicated that IVIg may attenuate the expression and activation of TLR9 in B cells from SLE patients, suggesting a novel additional mechanism of IVIg [1]. Another recent study showed that IVIg and F(ab)_2_ fragments of IVIg that lacks the Fc region inhibit TLR4-mediated activation of dendritic cells [29]. Our data reveals that treatment with IVIg significantly prevented ischemia-induced increased neuronal expressions of TLR2, TLR4 and TLR8. In addition, we have also found that ischemia-induced increased expressions of TLR adaptor and signalling proteins such as MyD88 and TRAF6 were prevented by IVIg treatment. These expression data indicate that IVIg may exert its beneficial effect by modulating the expression and activation of neuronal TLR2, TLR4 and TLR8.

It is well established that the activation of NF-κB, JNK and p38 MAPK pathways are implicated in TLR2, TLR4 and TLR8 signalling [2,30]. Our data show that IVIg treatments significantly attenuated the expression level of the phosphorylated forms of NF-κB p65, JNK, p38 MAPK and c-Jun in neurons following ischemic conditions. It is well known that NF-κB is a regulator of neuronal apoptosis in cerebral ischemia [31]. Similarly, p38 MAPK, JNK and c-Jun are known to contribute to neuronal cell death following ischemic condition [32]. Our data has shown that IVIg not only prevented ischemia-induced increased expression levels of TLR2, TLR4 and TLR8 and attenuated its signalling cascades but also ischemia-induced cleavage of apoptotic protease caspase-3.

Our data further suggests that IVIg protects neurons by modulating TLR expression and activation *in vitro* are also true for *in vivo*. We have previously reported that IVIg treatment reduces brain damage and improves neurological deficits after cerebral I/R injury [14]. In addition, we have shown that IVIg reduces ischemic stroke-induced infiltration of leukocytes and protects against endothelial dysfunction [27]. We also found evidence that the neuroprotective effects of IVIg are associated with a significant reduction in the levels of NLRP inflammasome proteins as well as precursors of both IL-1β and IL-18 in a mouse model of focal ischemic stroke [26]. Here, we show that the increased expression levels of TLR2, TLR4 and TLR8, adaptor protein MyD88, signalling protein TRAF6, TLR downstream signalling proteins such as NF-κB and MAPKs were significantly prevented in IVIg-treated animals compared to control animals following stroke.

The receptor for advanced glycation endproducts (RAGE) is a member of the immunoglobulin superfamily that is located on the plasma membrane and activated by a variety of ligands, including advanced glycation endproducts (AGEs) and HMGB1 [22,23]. Similar to the TLRs, ligand binding to RAGE leads to the activation of several intracellular inflammatory pathways, including NF-κB, JNK and p38 MAPK [33]. We have recently shown that RAGE-deficient mice were protected from ischemic stroke and HMGB1 promotes stroke-induced neuronal cell death by activating signalling cascades associated with RAGE and TLR activation [23]. Experimental evidence suggests that HMGB1 is released in large amounts into the extracellular space immediately after an ischemic brain insult and subsequently induces an inflammatory reaction via the activation of the TLRs and RAGE in affected brain tissues [23]. We have also demonstrated that recombinant soluble RAGE (sRAGE), acting as a decoy receptor for HMGB1, significantly reduced the infiltration of immune cells and improved the outcome of injury in mice subjected to I/R as well as protecting cultured neurons against ischemic cell death [23]. Our current data show that HMGB1 treatment increases TLR2, TLR4 and RAGE expression levels, and IVIg significantly prevented HMGB1-induced increases of these proteins. Similarly, HMGB1-induced MyD88 and TRAF6 expressions were also significantly prevented with IVIg treatment. Furthermore, IVIg significantly prevented HMGB1-induced increases of TLR/RAGE signalling cascade proteins such as NF-κB-p-p65, p-JNK, p-p38 MAPK and p-c-Jun.

## Conclusions

Our data establish that IVIg targets expression and activation of TLR and RAGE pathway components as well as protecting neurons against HMGB1-mediated neuronal cell death in ischemic stroke.

## Abbreviations

AGE: advanced glycation endproducts
ANOVA: one-way analysis of variance
Bcl-2: B-cell lymphoma 2
BSA: bovine serum albumin
CCA: common carotid artery
DAMPs: damage-associated molecular patterns
ECA: external carotid artery
GD: glucose deprivation
HMGB1: high-mobility group box 1
I/R: ischemia/reperfusion
ICA: internal carotid artery
IFN: interferon
IKKε: inhibitor of nuclear factor kappa-B kinase subunit epsilon
IVIg: intravenous immunoglobulin
IRAK4: interleukin-1 receptor-associated kinase 4
JNK: c-Jun N-terminal kinase
LPS: lipopolysaccharide
MAPK: mitogen-activated protein kinase
MCA: middle cerebral artery
MyD88: myeloid differentiation primary response gene (88)
NF-κB: nuclear factor kappa B
OGD: oxygen and glucose deprivation
PAMPs: pathogen-associated molecular patterns
PRRs: pattern recognition receptors
RAGE: receptor for advanced glycation endproducts
SLE: systemic lupus erythematosus
sRAGE: soluble RAGE
TBK1: TANK-Binding kinase 1
TIR: toll/interleukin-1 receptor
TLRs: toll-like receptors
TNF: tumour necrosis factor
TRAF6: TNF receptor associated factor 6
TRIF: TIR-domain-containing adapter protein inducing IFN-β.
